# Non-Vertical Exposures to HIV, HBV and HCV Infection in Children and Adolescents—Risk of Infection, Standards of Care and Postexposure Prophylaxis

**DOI:** 10.3390/pediatric13040067

**Published:** 2021-10-13

**Authors:** Anna Tomasik, Maria Pokorska-Śpiewak, Magdalena Marczyńska

**Affiliations:** 1 Doctoral School, Medical University of Warsaw, Żwirki i Wigury, 02-091 Warsaw, Poland; 2 Department of Children’s Infectious Diseases, Medical University of Warsaw, Wolska 37, 01-201 Warsaw, Poland; mpspiewak@gmail.com (M.P.-Ś.); magdalena.marczynska@wum.edu.pl (M.M.); 3 Regional Hospital of Infectious Diseases in Warsaw, 01-201 Warsaw, Poland

**Keywords:** postexposure prophylaxis, HIV, HBV, HCV, non-vertical exposure

## Abstract

Introduction: in the review, we aimed to present current knowledge about the risk of infection, standards of care, and postexposure prophylaxis (PEP) in pediatric patients after non-vertical exposures to HIV, HBV, and HCV infection. Materials and Methods: the latest available literature and recommendations of Centers for Disease Control and Prevention (CDC), World Health Organization (WHO), European recommendations for the management of HIV and administration of non-occupational PEP, and Polish AIDS Society were reviewed. Results: the majority of cases of non-vertical exposure to blood-borne viruses in the pediatric population consist of sexual exposition and injection with unsterilized sharp objects (usually needlestick injuries). The risk HIV, HBV, and HCV transmission depend on several factors, and each exposure should be evaluated individually with consideration of the patient’s medical history. It is crucial to start antiretroviral therapy within 48 h from exposure. Treatment is continued for 28 days, and a 3-drugs regiment is recommended in the majority of cases. Decisions on hepatitis B and tetanus PEP are based on a history of vaccination. There is no PEP for hepatitis C infection, follow-up testing aims for early identification of disease and consideration of treatment options. Conclusion: all children after the non-vertical exposure to HIV, HBV, and HCV infection should be evaluated by the Infectious Disease specialist as soon as possible after the incident and qualified to post-exposure prophylaxis. Systematic diagnostic and follow-up on children after significant needlestick exposure should be maintained. Children after sexual exposure need a multidisciplinary approach. Response to reported event must be rapid and treatment must be comprehensive.

## 1. Introduction

The majority of cases of non-vertical exposure to human immunodeficiency virus (HIV), hepatitis B virus (HBV), and hepatitis C virus (HCV) in the pediatric population consist of sexual exposure and injection with unsterilized sharp objects (usually needlestick injuries). In children, the predominant mode of HIV infection is vertical, from mother to child, although the virus can also be spread by sexual transmission and by blood [1]. The principle modes of HBV and HCV transmission are percutaneous, sexual, and perinatal exposures [2]. Data on the infectivity and postexposure prophylaxis in horizontal expositions to blood-borne infections in children are limited, consisting mainly of observational studies and case reports. Therefore, statistical data on the risk of infection and guidelines for antiretroviral drug use are mainly extrapolated from occupational expositions and prophylaxis used in vertical expositions. It is due to the fact that these situations rarely occur in clinical practice, and even the dedicated departments see only a few of those patients per year. Non-vertical exposures to blood-borne infections are associated with high anxiety among parents and pediatric patients, considering that up to 6 months are generally required to exclude infection. In the review, we aimed to present current knowledge about the risk of infection, standards of care, and postexposure prophylaxis (PEP) in pediatric patients after non-vertical exposures to HIV, HBV, and HCV infection.

## 2. Materials and Methods

The latest available literature, recommendations of the Centers for Disease Control and Prevention, World Health Organization, European recommendations for the management of HIV and administration of non-occupational PEP, and Polish AIDS Society were reviewed using PubMed and Medline. The following keywords were searched in these databases: postexposure prophylaxis, HIV, HCV, HBV, children, adolescents, non-vertical exposure.

## 3. Results

### 3.1. Estimated Infection Risk after Needlestick Injuries

The risk of blood-borne infection transmission after needlestick injury depends on several factors. Depth of penetration of the needle, presence of visible blood in the syringe, time passed since the needle was used, initiation of postexposure prophylaxis (PEP), and in case of HBV infection, the immunization status of the child. All of the blood-borne viruses: HBV, HCV, and HIV, can survive outside the human body. The virus vitality is influenced by virus concentration, the volume of blood, temperature variation, exposure to sunlight, and humidity [3,4]. Studies confirm that the risk of seroconversion to HIV, HBV, or HCV from a community-acquired needlestick injury is low [5,6,7,8,9]. The disparity between virus survival tested in laboratory conditions and transmission rate highlights the difficulty in extrapolating in vitro experiments to real-life scenarios. The lack of a well-established culture system or animal models has particularly impeded the evaluation of HCV infectiveness [3,10,11,12,13,14]. The risk of virus transmission depends on the prevalence of HIV, HBV, and HCV in the population. Thus, epidemiological data from a given country are crucial for clinical assessment and initiating PEP.

#### 3.1.1. HIV

The transmission rate of occupationally acquired HIV after needlestick injury is 0.3% (1 in 300 chance) [1,15]. This risk can increase up to 5% (1 in 20 chance) if the needle is contaminated with the blood of an HIV-positive patient with high viral load (early infection or terminal disease stage), the injection is deep with lots of blood and procedure involved patients vein or artery [15]. To estimate the potential risk of transmission from the discarded needles, the prevalence of injection drug use and HIV in the community where the incident took place should be considered [16]. However, HIV acquisition after exposure to dried blood found on syringes was not observed [17]. Only a few studies of pediatric patients after needlestick were published up to date. An observational study with the biggest sample size (274 children) was conducted in Canada over 19 years. The follow-up was possible for 186 children, who were tested for HIV after 6 months. No seroconversion was observed, and only 30% of those children received PEP [18]. The high and low-risk scenarios for HIV acquisition after needlestick injury are presented in Table 1.

#### 3.1.2. HBV

HBV is resistant to environmental changes and dried at room temperature remains infectious for at least one week. It can also survive on environmental surfaces that are not visibly contaminated with blood [19]. Numerous cases of environmental transmission of HBV have been reported. Household contacts are at a particularly high risk of acquiring the infection. Transmission can occur through blood-contaminated objects such as toothbrushes, razor blades, even rarely in association with human bites [2,20]. The efficiency of infectivity is due to the high resistance of the virus to environmental conditions and its high concentration in blood [21]. The risk of infection following the needle stick injury varies from 2% (when the source is anti-HBe negative) to 40% (when the source is anti-HBe positive). The presence of HBe antigen correlates with the replication level and infectivity [22]. Fortunately, HBV infection is vaccine-preventable. In the countries where children are routinely vaccinated against HBV, the majority of cases result in immunity to the disease. The vaccine is highly effective in protecting against HBV infection, and with high immunization coverage, the number of active carriers is also decreasing. No routine booster doses are recommended for healthy children. Studies indicate that immunologic memory lasts for a minimum of 30 years for healthy individuals who completed vaccination with 3 dosages, and cellular immunity remains even in the event of antibody level decrease [23,24].

#### 3.1.3. HCV

HCV infection was associated mainly with blood transfusion until the implementation of blood products screening. Nowadays, in developed countries, intravenous drug use has become the major route of HCV transmission [25]. The risk of transmission of HCV is significantly greater than the risk of HIV transmission after blood-borne exposure. HCV acquisition risk is estimated to be 1.8% after occupational exposure [16]. However, needles discarded in the parks and playgrounds are affected by temperature and humidity changes for an undetermined time—hence the risk of infection should be lower than in the hospital environment. Survival of HCV in syringes for prolonged periods was tested experimentally. Researchers found that in laboratory conditions, HCV endurance was influenced by syringe shape and time of exposure to changing environmental conditions—syringes with detachable needles seemed more likely to transmit HCV [26]. In the study conducted in Canada, 159 children after needlestick injury were tested 6-months after the incident for HCV, and no seroconversion was observed [18]. Up to date there was one documented case of HCV acquisition after community-acquired needlestick injury [14]. Unfortunately, there is no pre-exposure prophylaxis available against HCV infection. However, recently very effective and safe new therapies for chronic HCV infection based on direct-acting antivirals (DAA) were approved for children.

### 3.2. Estimated Infection Risk after Sexual Exposition 

In this article, we focus on sexual assault, which requires emergency treatment and consideration for initiating PEP. The data on the risk of HIV, HBV, and HCV infection after sexual exposition cited in the following paragraphs are extrapolated from studies on the adult population due to the fact that such data regarding children are scarce or unobtainable. The situation of sexually active adolescents who engage in frequent sexual contacts and high-risk behaviors and would require consideration for pre-exposure-prophylaxis is a separate issue that exceeds extend of this review and will not be addressed.

#### 3.2.1. HIV

The risk of HIV infection after sexual assault depends on the type of sexual contact, presence of sexually transmitted diseases (STDs), HIV status of the source, genitourinary trauma, and circumcision status [27]. The risk of the infection from sexual exposure is low for oral sex but substantial in the case of receptive anal intercourse (138 infections per 10,000 exposures) [28]. In the study by Mastro and de Vincenzi [29], the risk estimated for receptive vaginal intercourse without a condom was 0.08% and for insertive vaginal intercourse 0.04%. The higher risk scenarios of HIV acquisition include mucosal trauma, bleeding, and absence of barrier protection [Table 2]. 

#### 3.2.2. HBV

HBV infection as a sexually transmitted disease is well documented. Moderate viral concentrations were found in semen, vaginal secretions, and saliva of the HBV-infected individuals [2,20]. Sexual transmission of HBV has been associated with multiple sexual partners; history of another sexually transmitted infection (STD); and anal intercourse [30], which is usually more traumatic than vaginal and can result in exposure to blood [31]. HBV infection is also very common among individuals in contact with sex workers [32]. The risk of transmission is high for those with no history of vaccination. 

#### 3.2.3. HCV

HCV can be transmitted by sexual contact. However, the efficiency of that transmission route is controversial. [33]. Studies on the infectiveness of HCV virus yielded mixed results. HCV RNA was present in semen and vaginal secretions. However, HCV RNA detected in semen of HCV viremic men was of low titer [34]. High-risk scenarios for HCV transmission by sexual contact are HIV infection, multiple sexual partners, traumatizing mucous membranes during sexual practices, presence of other genital infections, and men having sex with men [33]. In the study conducted on monogamous heterosexual couples where one partner had chronic HCV, the rate of transmission to a discordant partner was extremely low [35]. For individuals with chronic HCV infection, the estimated risk of sexual transmission of the virus is 0–0.6% per year for those in a monogamous relationship and 1% per year for those with multiple sexual partners [33]. 

### 3.3. Standards of Care

All children after non-vertical exposure to HIV, HBV, and HCV infection should be consulted by an infectious disease specialist as soon as possible after the incident. Precise history taking allows estimating the infection risk and implementation of PEP. The initial evaluation includes the patient’s baseline serological status testing for HIV, HBV, HCV as shown in [Table 3]. 

The recommendations for follow-up visits are formed locally. However, they are quite similar among European countries and even the USA. Most of the recommendations are based on the same studies, and literature reviews as only a few were published on the topic of non-vertical expositions to HIV, HBV, and HCV. We decided to present a comparison of guidelines formed by CDC and Polish AIDS Society—as an example of European recommendations [Table 4 and Table 5].

The basic follow-up visits schedule requires up to 3 months to determine if the patient was infected with HIV. The exceptional case of acquiring HCV and HIV simultaneously can delay HIV seroconversion and requires additional testing for HIV 6 months after the exposition. The golden standard is anti-HIV antibodies and p24 antigen testing on each visit. The follow-up testing for individuals susceptible to HBV and HCV at baseline can take up to 6 months, depending on the type of tests available. If the HCV-RNA test can be performed 4 weeks after exposition together with alanine aminotransferase (ALT) level and is negative, no further testing is indicated according to Polish AIDS Society recommendations [Table 4]. However, HCV_RNA test might not be easily available thus the alternative testing requires HCV antibody and ALT level testing 6 months after the exposition.

Polish AIDS Society recommendations schedule more follow-up visits than the CDC guidelines. The reason is close patient monitoring after initiating ARV therapy. The visit 2 weeks after the incident allows us to test early for toxic side effects of the drugs. The patients have a chance to talk about observed side-effects and ask questions about the therapy that they might not have understood on the initial visit due to the stress and trauma. 

Close follow-up is necessary for monitoring adherence to therapy, toxic side effects of drugs, and to complete serial testing for HIV, HBV, and HCV infection with the serological window period in consideration. If testing of the source is possible and his/her status is cleared, the follow-up testing of the exposed patient can be discontinued. 

Time is crucial as PEP has to be initiated within 48 h after the incident (in case of high-risk exposures no later than 72 h). The effectiveness of PEP diminishes with time starting 2 h after the incident [16]. PEP with antiretroviral drugs is continued for 28 days, and a 3-drug regimen is recommended in the majority of cases [Table 6 and Table 7]. 

**Figure 1:**
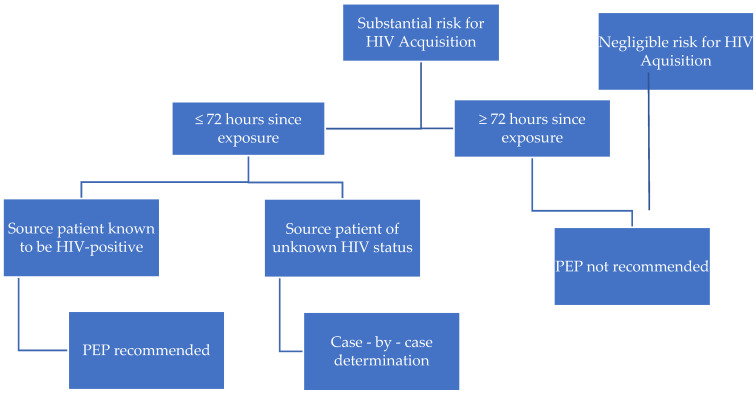
Algorithm for evaluation and treatment of possible non-vertical exposure to HIV [27].

The same antiretroviral drugs, which are proposed in CDC and WHO guidelines are recommended as the first line treatment in most of the countries around the world [27,37,38,39]. The differences are the result of product registration for children of a certain age in each country. Clinicians are also limited in ARV choice for the youngest children and infants by the administration forms of the drug available on the market. The youngest patients need to receive oral solutions, not tablets, which is the reason for proposing lamivudine, zidovudine, and lopinavir/ritonavir regimen as a first-line therapy for children <12 years old in Poland [Table 6]. The basic algorithm for initiating HIV PEP according to CDC guidelines is presented in [Figure 1] and the substantial and negligible risk scenarios in Table 8. We stress that each situation should be considered and evaluated individually.

The most commonly reported side effects of antiretroviral therapy are nausea, vomiting, diarrhea, and fatigue. Follow-up visits allow reporting and ameliorating specific side effects, which should improve adherence to the therapy. Patients must be educated to recognize early symptoms associated with primary HIV infection and instructed to report for evaluation if these occur during the follow-up period [Table 9] [39]

Due to the absence of efficient PEP for HCV infection, the recommendations suggest baseline and follow-up testing of HCV infection. This aims for early identification of disease and consideration of treatment options. Hepatitis B PEP implementation is based on the history of vaccination and post-vaccination anti-HBs level (greater than 10 mIU/mL is considered protective). Patients who are not immunized against hepatitis B patients should receive hepatitis B immunoglobulin, administered within the first 24 h after exposure. It is used in combination with active immunization against hepatitis B with vaccine in the scheme of 0–1–6 months (3 doses). A booster dose should be administrated to patients vaccinated against hepatitis B with an anti-HBs level lower than 10 mIU/mL. Similarly, in case of needlestick injuries, immunization history in regard to tetanus has to be reviewed.

Pediatric patients should be evaluated for other exposure-associated health risks such as sexually transmitted infections. Forensic examination and reporting to local authorities should be a priority in case of sexual assault. Girls should be consulted by a gynecologist and considered for emergency contraception. Additionally, all of these patients should be consulted by a psychologist or psychiatrist.

## 4. Conclusions

Each case of non-vertical exposure to blood-borne viruses needs to be evaluated individually. Even though the risk of infection with blood-borne viruses after needlestick injury seems to be low, it remains a possibility in high-risk scenarios. Therefore, systematic diagnostic and follow-up in children after significant needlestick exposure should be maintained. There is a need for more studies to be conducted on the topic to create guidelines based on solid evidence, as the available literature is becoming quite old. Children after sexual exposure need a multidisciplinary approach and professional consultations of infectious disease specialists, pediatric obstetric, forensic, and emergency medicine specialists, and psychologist or psychiatrist, who are required to meet the challenge. Due to complex and sensitive nature of children and adolescents’ sexual expositions, the response to the reported event must be rapid, and treatment must be comprehensive.

## Figures and Tables

**Table 1:** Risk of HIV acquisition after needlestick injury [11].

High Risk of HIV Acquisition	Negligible Risk of HIV Acquisition
HIV—positive source High prevalence of HIV in injection drug users in the region Large lumen needle with visible blood in the needle or syringe Injection of blood	No visible blood in the needle or syringe Low prevalence of HIV in injection drug users in the region Exposure to environmental conditions—drying, heat, and freezing since use Superficial injury

**Table 2:** Factors influencing the risk of HIV transmission during sexual exposure according to CDC guidelines [27].

Higher Risk of HIV Acquisition	Lower Risk of HIV Acquisition
Presence of other sexually transmitted diseases Acute/late-stage HIV infection of the source Receptive anal intercourse Mucosal trauma/bleeding	Condom use Male circumcision

**Table 3:** Patient’s initial testing universal recommendations [27,36].

Exposure	Tests
HIV	HIV Ab/Ag (IV generation test)
HBV	HBs Ag HBc Ab HBs Ab (in previously vaccinated patients)
HCV	HCV Ab
Sexual exposure (additional tests)	Syphilis serology (e.x. VDRL) Pregnancy test

Ag—antigen; Ab—antibody; VDRL—Veneral Diseases Research Laboratory.

**Table 4:** Schedule of follow-up visits according to recommendations of Polish AIDS Society, 2021 [36].

Exposure	Initial Visit	2 Weeks	4 Weeks	8 Weeks	24 Weeks
All non-vertical exposures	HIV Ab/Ag HBs Ag HBc Ab HBs Ab HCV Ab CBC ALT AST Creatinine	CBC ALT AST Creatinine	HIV Ab/Ag HCV-RNA ALT	HIV Ab/Ag	HBs Ag ^b^ HBc Ab ^b^ HCV Ab ^c^ ALT ^c^
Sexual exposure (additional tests)	Syphilis serology (e.x. VDRL) Pregnancy test			Syphilis serology (e.x. VDRL)	

CBC—complete blood count; ALT—alanine aminotransferase; AST—aspartate aminotransferase; Ab—antibody; Ag-antigen; VDRL—Veneral Diseases Research Laboratory. ^b^ testing depends on the history of vaccination and post-vaccination anti-HBs level. ^c^ if HCV-RNA test at 4 weeks was unavailable.

**Table 5:** Schedule of follow-up visits according to CDC guidelines [27].

Exposure	Initial Visit	4–6 Weeks	12 Weeks	24 Weeks
All non-vertical exposures	HIV Ab/Ag HBs Ag HBc Ab HBs Ab HCV Ab ALT AST Creatinine	HIV Ab/Ag ALT AST Creatinine	HIV Ab/Ag	HIV Ab/Ag ^a^ HBs Ag ^b^ HBc Ab ^b^ HBs Ab ^b^ HCV Ab ^c^
Sexual exposure (additional tests)	Syphilis serology (e.x. VDRL) Pregnancy test	Syphilis serology (e.x. VDRL) Pregnancy test		

ALT—alanine aminotransferase; AST—aspartate aminotransferase; Ab—antibody; Ag-antigen; VDRL—Veneral Diseases Research Laboratory. ^a^ only if Hepatitis C infection was aquired during the original exposure; delayed HIV seroconversion was has been seen in persons who simultaneously acquire HIV and HCV. ^b^ if exposed person susceptible to Hepatitis B at baseline. ^c^ if exposed person susceptible to Hepatitis C at baseline.

**Table 6:** Postexposure prophylaxis—first choice ARV drug regimens for pediatric patients according to recommendations of the Polish AIDS Society [36].

Children under 12 Years Old	Children over 12 Years Old
1. Zidovudine: 9 mg/kg twice a day (maximum 2 × 300 mg) 2. Lamivudine: 4 mg/kg twice a day (maximum 2 × 150 mg) 3. Lopinavir/ritonavir: Lopinavir: 10 mg/kg twice a day Ritonavir: 2.5 mg/kg twice a day (maximum dose 2 × 400/100 mg)	Emtricitabine + Tenofovir: 200/245 mg once daily Darunavir: 800 mg once daily Ritonavir 100 mg once daily OR Emtricitabine + Tenofovir: 200/245 mg once daily Raltegravir: 400 mg twice a day

**Table 7:** Postexposure prophylaxis—ARV drug regimens for pediatric patients according to CDC guidelines [27].

Children Aged 2–12 Years Old	Adolescents Aged 13 Years Old and Older
Prefered: Emtricitabine + Tenofovir Raltegravil Alternative: Zidovudine Lamivudine Raltegravir or Lopinavir/ritonavir With drugs dosed to age and weight	Preferred: Emtricitabine 200 mg + Tenofovir DF 300 mg Raltegravir: 400 mg twice a day or Dolutegravir 50 mg once daily Alternative: Emtricitabine 200 mg + Tenofovir DF 300 mg Darunavir: 800 mg once daily Ritonavir 100 mg once daily

**Table 8:** Substantial and negligible risk scenarios for HIV Acquisition according to CDC guidelines [27].

Substantial Risk for HIV Acquisition	Negligible Risk for HIV Acquisition
Exposure of: vagina, rectum, eye, mouth, or other mucous membrane, nonintact skin, or percutaneous contact With: blood, semen, vaginal secretions, rectal secretions, breast milk, or any body fluid that is visibly contaminated with blood When: The source is known to be HIV-positive	Exposure of: vagina, rectum, eye, mouth, or other mucous membrane, intact or nonintact skin, or percutaneous contact With: urine, nasal secretions, saliva, sweat, or tears if not visibly contaminated with blood Regardless of the known or suspected HIV status of the source

**Table 9:** Clinical symptoms of primary HIV infection [39].

Clinical Signs and Symptoms of Primary HIV Infection
Fever Fatigue Myalgia Skin rash Headaches Pharyngitis Cervical adenopathy Arthralgia Night sweats Diarrhea

## Data Availability

Not applicable.
